# Protection Induced by Vaccination with Recombinant Baculovirus and Virus-like Particles Expressing *Toxoplasma gondii* Rhoptry Protein 18

**DOI:** 10.3390/vaccines10101588

**Published:** 2022-09-22

**Authors:** Keon-Woong Yoon, Ki-Back Chu, Hae-Ji Kang, Min-Ju Kim, Gi-Deok Eom, Jie Mao, Su-Hwa Lee, Md Atique Ahmed, Fu-Shi Quan

**Affiliations:** 1 Department of Biomedical Science, Graduate School, Kyung Hee University, Seoul 02447, Korea; 2 Medical Research Center for Bioreaction to Reactive Oxygen Species and Biomedical Science Institute, School of Medicine, Graduate School, Kyung Hee University, Seoul 02447, Korea; 3 Department of Medical Zoology, School of Medicine, Kyung Hee University, Seoul 02447, Korea; 4 ICMR-Regional Medical Research Centre, NE Region, Dibrugarh 786010, Assam, India

**Keywords:** baculovirus, virus-like particle, *Toxoplasma gondii*, ROP18, vaccine

## Abstract

Heterologous immunization is garnering attention as a promising strategy to improve vaccine efficacy. Vaccines based on recombinant baculovirus (rBV) and virus-like particle (VLP) are safe for use, but heterologous immunization studies incorporating these two vaccine platforms remain unreported to date. Oral immunization is the simplest, most convenient, and safest means for mass immunization. In the present study, mice were immunized with the *Toxoplasma gondii* rhoptry protein 18 (ROP18)-expressing rBVs (rBVs-ROP18) and VLPs (VLPs-ROP18) via oral, intranasal, and intramuscular (IM) routes to evaluate the protection elicited against the intracellular parasite *T. gondii* ME49 strain. Overall, boost immunization with VLPs-ROP18 induced a significant increase in *T. gondii*-specific antibody response in all three immunization routes. Parasite-specific mucosal and cerebral antibody responses were observed from all immunization groups, but the highest mucosal IgA response was detected from the intestines of orally immunized mice. Antibody-secreting cell (ASC), CD8^+^ T cell, and germinal center B cell responses were strikingly similar across all three immunization groups. Oral immunization significantly reduced pro-inflammatory cytokine IL-6 in the brains as well as that by IN and IM. Importantly, all of the immunized mice survived against lethal challenge infections where body weight loss was negligible from all three immunizations. These results demonstrated that protection induced against *T. gondii* by oral rBV-VLP immunization regimen is just as effective as IN or IM immunizations.

## 1. Introduction

Parenteral immunization, such as intramuscular (IM) vaccine delivery, is one of the most commonly used methods of immunization. However, IM inoculation of vaccine components rarely induces mucosal immunity and requires multiple immunizations to mount adequate antibody responses in vaccinees [1]. For this reason, alternative vaccination routes such as oral and intranasal (IN) inoculation of vaccines are actively being investigated. While both oral and IN routes can elicit a strong mucosal immune response, adverse events have been reported from the latter. In fact, Guillain-Barre syndrome, encephalomyelitis, Bell’s palsy, and other neuropathological vaccine-associated adverse events were reported from individuals receiving intranasal influenza vaccines [2]. Oral immunization, though not as effective as the IN route in terms of immunity induction, is generally perceived to be safe, easy to administer, and able to stimulate both systemic and mucosal immune responses [3,4]. Therefore, developing effective vaccines that are administered through the oral route could yield promising results.

*Toxoplasma gondii* is a coccidium capable of parasitizing any warm-blooded animals to cause the zoonotic disease toxoplasmosis [5]. Unfortunately, none of the currently available therapeutic options can effectively eliminate the tissue-dwelling cysts of *T. gondii*. This situation, further exacerbated by the emergence of drug-resilient *T. gondii* strains and adverse side effects of the drugs, has created an impetus for vaccine development [6,7]. While a live-attenuated vaccine based on the *T. gondii* S48 strain is commercially available, its application is limited to ewes and an effective toxoplasmosis vaccine for humans remains under development. To date, in an attempt to develop a highly efficacious human toxoplasmosis vaccine, a plethora of *T. gondii* antigens expressed using various vaccine platforms have been explored. Despite being inherently safe in numerous eukaryotic organisms [8], baculovirus-based vaccines are underutilized in *T. gondii* vaccine research. Currently, only a few studies investigated the potential of baculovirus-based vaccines against *T. gondii* [9,10]. Previously, we have also demonstrated that immunizing mice with the recombinant baculovirus (rBV) vaccines expressing the rhoptry protein 4 (ROP4) of *T. gondii* can induce protection [11]. Nonetheless, ROP4-rBV immunizations neither completely inhibited cyst formation nor prevented bodyweight loss following *T. gondii* ME49 challenge infection, which signified the need for further improvements.

Some strategies for enhancing vaccine efficacy include adjusting the immunization regimen, adjuvant use, and many others. To address the limitation of our previous study, we chose to increase the number of vaccinations and employ the heterologous immunization strategy. Our previous study using VLP vaccines demonstrated that the number of immunizations is correlated with *T. gondii* cyst burden reduction [12]. Similarly, cyst formation was significantly reduced in mice subjected to heterologous immunization with differing viral-vectored vaccines [13]. Recently, it was reported that ROP18 antigens are highly conserved across several lineages of *T. gondii* and are involved in the parasite’s immune evasion process in intermediate hosts [14,15]. Based on these findings, we hypothesized that rBVs expressing the ROP18 antigen in place of ROP4 may confer enhanced protection, especially in combination with the changes to the immunization regimen. Here, mice were heterologously immunized three times with ROP18-expressing rBV or VLP vaccines, and the overall protection induced by this immunization strategy upon *T. gondii* ME49 challenge infection was assessed. We also compared the protective efficacy elicited by different administration routes to optimize the immunization strategy.

## 2. Materials and Methods

### 2.1. Animal Ethics

Six-week-old female BALB/c mice (*n* = 6 per group) were purchased from NARA Biotech (Seoul, South Korea). Mice were maintained under specific-pathogen-free conditions in an approved facility. Mice were given easy access to food and water, along with a 12 h day and night time cycle. All experimental protocols were approved and performed according to the guidelines of Kyung Hee University IACUC (permit number: KHUASP (SE) 20-648).

### 2.2. Cells, Parasites, and Antibodies

Parasites and cells used in this study were maintained as previously described [16]. *T. gondii* ME49 strain was maintained in BALB/c mice by serial passaging. Spodoptera frugiperda (Sf9) cells were cultured in spinner flasks with serum-free SF900II medium (Invitrogen, Carlsbad, CA, USA) at 27 °C, 130–135 rpm. Horseradish peroxidase (HRP)-conjugated goat anti-mouse IgG and IgA secondary antibodies were purchased from Southern Biotech (Birmingham, AL, USA).

### 2.3. Recombinant Baculovirus (rBV) and VLP Vaccine Generation

rBVs-ROP18 were prepared as previously described [17]. Briefly, the PCR-amplified ROP18 gene was cloned into pFastBac vector. DH10Bac competent cells were transformed with the recombinant pFastBac vector and bacmid DNA was acquired. After transfecting Sf9 cells with the bacmid DNA, supernatants containing the ROP18-rBVs were carefully collected. rBVs-ROP18 were either stored at −80 °C for later use or used to generate VLPs-ROP18. For VLP production, Sf9 cells were co-infected with influenza M1-rBVs and ROP18-rBVs as previously described [17]. ROP18 expressions in rBVs and VLPs were confirmed by ELISA and transmission electron microscope (TEM). Serially diluted rBVs and VLPs were coated in immunoplates, and the mouse polyclonal *T. gondii* antibody was used to perform ELISA as described [11].

### 2.4. Vaccination and Challenge Infection Schedule

A total of 30 mice were divided into 5 groups (*n* = 6 per group) as follows: unimmunized and uninfected (Naïve), unimmunized mice subjected to *T. gondii* ME49 infection (Naïve+Cha), oral, IN, and IM immunization groups. Mice in the immunization groups received 5 × 10^5^ pfu ROP18-rBVs through respective administration routes for the first and second immunizations at a 4-week interval. For the third immunization, 50 μg of ROP18-VLPs were inoculated into each mouse. Four weeks after the final immunization, mice were orally challenge-infected with a lethal dose of ME49 cysts (450 cysts per mouse). All mice were monitored daily to record body weight changes and survival rates. At 35 days post-infection (dpi), all of the mice were sacrificed for organ sampling and ex vivo immunological analysis purposes.

### 2.5. Sample Preparation

Blood samples were collected by retro-orbital plexus puncture 3 weeks after each immunization. Mice were sacrificed at 35 dpi to acquire brain, mesenteric lymph nodes (MLN), spleen, and intestines following the method previously described [18]. Spleens were carefully homogenized using frosted microscope glass slides for splenocyte acquisition. Red blood cells (RBCs) were lysed by incubating cells with the RBC lysis buffer at room temperature for 10 min. To wash and remove the RBC debris, cells were washed twice with PBS and centrifuged at 2000 rpm, 4 °C for 5 min. Splenocytes were counted and used for antibody-secreting cell assays. Brain tissues were homogenized and centrifuged. The supernatant fractions were harvested and stored at −20 °C for cytokine assays, while the sedimented pellets were used for cyst quantification. The intestines were cut open along the vertical axis and immersed in 500 μL of PBS. The intestine samples were incubated at 37 °C for 1 h and then centrifuged at 5000 rpm for 10 min. Supernatants were collected and stored at −20 °C until use. All samples were individually processed.

### 2.6. Antibody Reaction

*T. gondii*-specific antibody responses were determined from serum, brain, and mucosal tissues using enzyme-linked immunosorbent assay (ELISA) as previously described [18]. Briefly, *T. gondii* ME49 were coated with 100 μL of carbonate coating buffer (4 μg/mL) on a flat-bottom 96 well immunoplate and incubated overnight at 4 °C. Plates were blocked with 0.2% gelatin dissolved in 0.1 M PBS with 0.05% Tween 20. Sera and mucosal samples diluted in PBS were used as primary antibodies (1:50 sera, 1:100 intestine, 1:3 vaginal sample dilution). After incubating the plates with primary antibodies for 1 h at 37 °C, HRP-conjugated goat anti-mouse IgG and IgA (diluted 1:2000 in PBS) were inoculated into each well and incubated at 37 °C for 1 h. O-phenylenediamine (OPD) substrate was dissolved in a citrate-phosphate buffer containing 0.03% H_2_O_2_ and used for color development. Optical density readings at 490 nm were measured using an ELISA reader (EZ Read 400, Biochrom Ltd., Cambridge, UK).

### 2.7. Antibody Secreting Cell (ASC) Response

Mice were sacrificed at 35 dpi and single cell suspensions of splenocytes were prepared as previously described [18]. After lysing the red blood cells, splenocytes (1 × 10^6^ cells/200 μL) isolated from each mouse were seeded into 96-well plates coated with *T. gondii* ME49 antigen (4 μg/mL). Plates were incubated at 37 °C, 5% CO2 for 4–5 days. After incubation, plates were briefly centrifuged at 2000 rpm, 4 °C, 5 min, and supernatants were aspirated. HPR-conjugated goat anti-mouse IgG and IgA secondary antibodies were added into respective wells and plates were incubated for 1 h, 37 °C. An OPD-based colorimetric assay was performed and OD 490 nm values were measured to determine ASC responses.

### 2.8. Flow Cytometry of Immune Cell Populations

Splenocytes and MLN cells were prepared for flow cytometry as previously described [12]. Splenocytes (1 × 10^6^ cells/mouse) and MLN cells (1 × 10^5^ cells/mouse) were stimulated ex vivo with *T. gondii* ME49 antigen (2 μg/mL) at 37 °C with 5% CO_2_ for 2 h. Fluorophore-conjugated CD3 (PE-Cy7), CD4 (FITC), CD8 (PE), GL7 (PE), and B220 (FITC) antibodies were purchased from BD Biosciences (Franklin Lakes, NJ, USA) and Thermo Fisher Scientific (Waltham, MA, USA) to assess the CD4^+^ T cell, CD8^+^ T cell, and germinal center (GC) B cell populations present in these lymphoid organs.

### 2.9. Inflammatory Cytokine Analysis

At 35 dpi, mice were sacrificed and brain tissues were homogenized to analyze pro-inflammatory cytokine production. IFN-γ and IL-6 levels in the brain tissues were evaluated using the BD OptEIA ELISA kit (BD Biosciences, Franklin Lakes, NJ, USA). Experiments were performed following the manufacturer’s instructions, and standard curves were generated to determine the concentration of each cytokine.

### 2.10. Parasite Burden

*T. gondii* ME49 cysts were isolated from the brain tissues as previously described [18]. Briefly, brain homogenates were mixed with Percoll density gradient solution and centrifuged at 12,100 rpm for 20 min. *T. gondii* cyst layer was carefully collected and resuspended in PBS. Cysts were placed on a slide glass and counted under a microscope (Leica DMi8, Leica, Wetzlar, Germany). Cysts were counted from 3 different fields of views per mice.

### 2.11. Statistical Analysis

All statistical analyses were performed using GraphPad Prism version 6 software (San Diego, CA, USA). All data were acquired on an individual basis and experiments were performed in triplicates. Data sets were expressed as mean ± SD. Statistical significance between groups was determined using a 2-way ANOVA with Tukey’s post hoc test to determine if the means were significantly different. *p*-values (* *p* < 0.05, ** *p* < 0.01, *** *p* < 0.001, and **** *p* < 0.0001) were used to determine statistical significance, which were denoted using asterisks.

## 3. Results

### 3.1. Characterization of Vaccines and Animal Experimental Schedule

**Figure 1:**
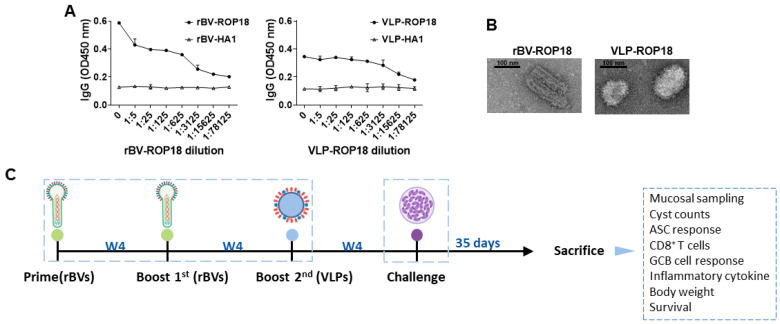
Characterization of rBVs and VLPs, and immunization schematic. Polyclonal *T. gondii* antibody was used to assess ROP18 proteins in rBVs and VLPs via ELISA (**A**). rBVs-ROP18 and VLPs-ROP18 were observed under a transmission electron microscope (TEM) (**B**). BALB/c mice were primed-boosted with rBVs-ROP18 and subsequently 2nd boosted with VLPs-ROP18 via oral, IN, and IM routes at regular intervals (**C**). All immunization, infection, sample collection schedules, immunological assay, and bodyweight with survival conducted were exactly followed as depicted above.

ROP18 in rBVs and VLPs was characterized by ELISA using polyclonal mouse anti-*T. gondii* antibody (Figure 1A). As indicated, polyclonal *T. gondii* sera successfully reacted with serially diluted ROP18 rBVs and VLPs while influenza hemagglutinin (HA1) from A/PR/8/34 showed no reactivity with the anti-*T. gondii* antibody. Rod-shaped rBVs and spherical shaped VLPs were observed by TEM (B). Vaccination, animal experiments, and immunological assay were performed as indicated (Figure 1C). 

### 3.2. Antibody Response in Immune Serum

**Figure 2:**
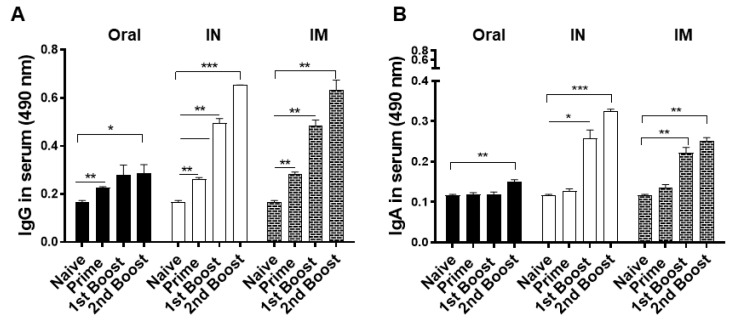
BALB/c mice were immunized with the vaccines as scheduled. Sera were collected from mice 3 weeks after each immunization and *T. gondii*-specific IgG (**A**) and IgA (**B**) antibody responses were measured by ELISA. Data are presented as mean ± SD and asterisks indicate statistical differences between groups. (* *p* < 0.1, ** *p* < 0.01, *** *p* < 0.001). All samples were acquired on an individual basis, and a total of three independent experiments were performed in triplicates.

*T. gondii*-specific IgG and IgA responses were measured from sera after each immunization. While IgG antibody inductions were comparable following prime immunization, noticeable differences were observed after the second immunization (Figure 2A). Specifically, IN and IM routes induced drastically higher IgG responses than the oral route. Overall, *T. gondii*-specific IgG responses between IN and IM routes were comparable at all serum collection time points. 

IgA response was similar to that of IgG response as indicated by the sharp increase in antibody levels following first boost immunization in the IN and IM immunization groups (Figure 2B). A marginal increase in antigen-specific IgA response was detected after second boost immunization from the oral immunization group. While a substantial increase in IgA antibodies was elicited by both IN and IM routes, antibody inductions occurred to a slightly greater extent in the former of the two.

### 3.3. ROP18-Expressing Vaccines Induce Parasite-Specific Antibody Responses in the Intestines and the Brain

**Figure 3:**
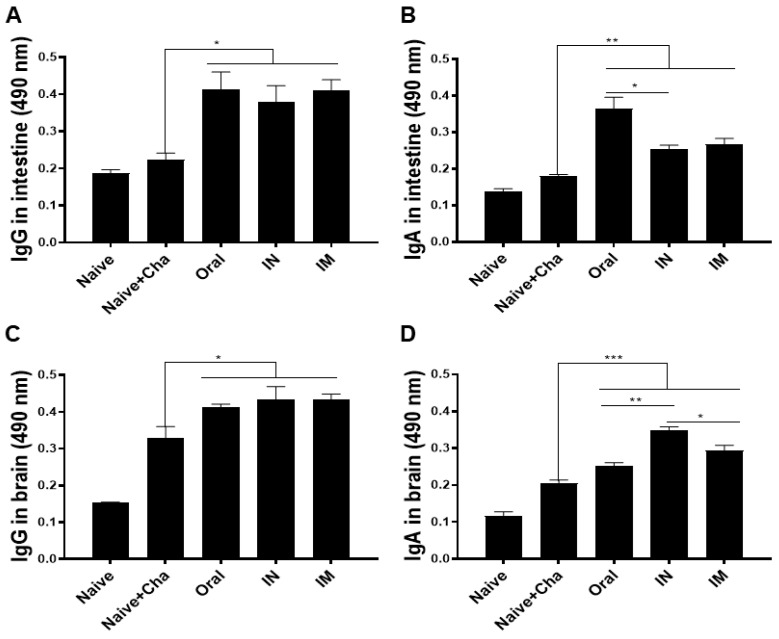
*T. gondii*-specific IgG and IgA antibody responses were observed by ELISA from the intestinal (**A**,**B**) and brain (**C**,**D**) tissue samples. Data are presented as mean ± SD and asterisks indicate statistical differences between groups. (* *p* < 0.1, ** *p* < 0.01, *** *p* < 0.001). All samples were acquired on an individual basis and a total of three independent experiments were performed in triplicates.

To evaluate the induction of mucosal immunity, antibody production in the intestines of mice was observed. Mice were sacrificed at 35 dpi and mucosal antibody responses of immunized groups were compared to that of the Naïve+Cha control group. *T. gondii*-specific intestinal IgG inductions were similar for all immunized mice, regardless of vaccine administration routes. Infecting unimmunized mice with *T. gondii* resulted in a negligible increase in intestinal IgG compared to naïve control (Figure 3A). Intestinal IgA responses were similar for the control groups. Incremental changes in *T. gondii*-specific IgA levels were detected from Naïve+Cha. Surprisingly, the highest IgA inductions were observed from orally immunized mice, whose levels were higher than those elicited by IN or IM immunization (Figure 3B). As with intestinal IgG, intracerebral IgG responses were enhanced following immunization. Heterologous immunization with the ROP18 vaccines resulted in significantly increased brain IgG levels compared to the control groups, irrespective of the vaccination routes (Figure 3C). An identical trend was also observed for the IgA response in the brain tissues. Mucosally administered ROP18 vaccines induced significantly greater IgA responses than IM administration (Figure 3D). 

### 3.4. Vaccination Induces Antibody-Secreting Cell Responses

**Figure 4:**
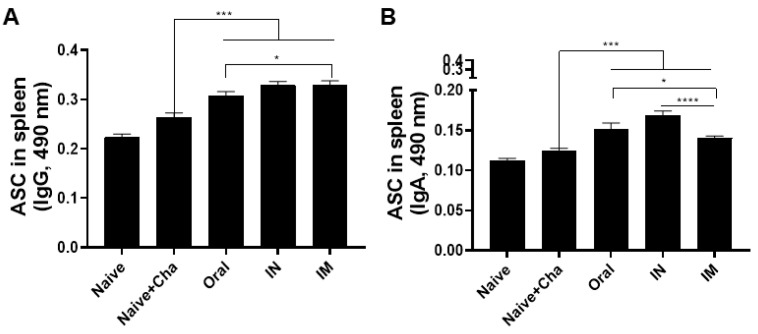
Splenocytes were co-cultured with *T. gondii* antigens and antibody-secreting cell responses were evaluated. *T. gondii*-specific IgG (**A**) and IgA (**B**) ASC responses were quantified by ELISA. Data are presented as mean ± SD and asterisks indicate statistical differences between groups. (* *p* < 0.05, *** *p* < 0.001, **** *p* < 0.0001). All samples were acquired on an individual basis and a total of three independent experiments were performed in triplicates.

To confirm the ASC responses, splenocytes from mice were collected and cultured for 5 days. A marked increase in the IgG response was observed in the splenocytes of immunized mice (Figure 4A). Noticeable increases in IgA ASC responses were detected following immunization, with IN immunization inducing the strongest response (Figure 4B). Consistent with the serum antibody responses, ASC IgG levels were elevated to a greater extent than the IgA in the immunized mice.

### 3.5. Activation of CD8^+^ T Cells and Germinal Center (GC) B Cells in the Spleen and MLN

**Figure 5:**
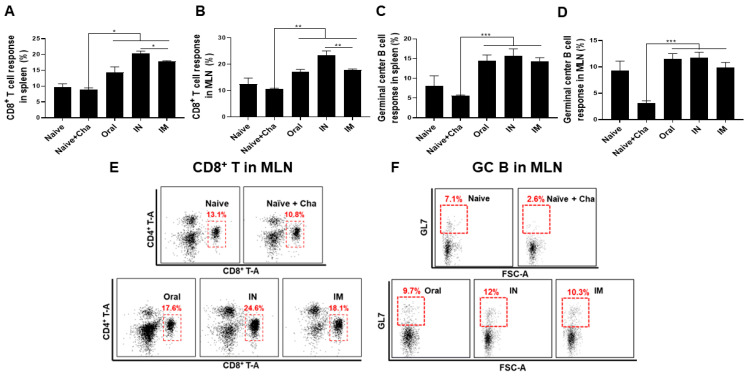
Mice were sacrificed at 35 dpi for sampling purposes. Flow cytometry was performed using the acquired splenocytes and MLN cells. CD8^+^ T cells from spleen (**A**) and MLN (**B**), as well as germinal center (GC) B cells from both spleen (**C**) and MLN (**D**) were detected after staining with appropriate fluorophore-conjugated antibodies. Scatterplots for CD8^+^ T cells and GC B cells from MLN were provided, with boxes indicating the gated CD8^+^ T cell population (**E**) and GC B cell population (**F**). Data are presented as mean ± SD and asterisks indicate statistical differences between groups. (* *p* < 0.1, ** *p* < 0.01, *** *p* < 0.001).

Flow cytometry was performed to evaluate the proliferation of CD8^+^ T cells in the mouse spleen and MLN. When infected with a lethal dose of *T. gondii* ME49, a negligible level of T cell depreciation occurred in the unimmunized control. Heterologous immunization ensured that mice retained high levels of CD8^+^ T cell populations necessary for protection in both spleen and MLN. Specifically, when compared to the Naïve+Cha group, the proportion of splenic CD8^+^ T cells was enhanced by 1.6-fold, 2.3-fold, and 2-fold in the oral, IN, and IM groups, each, respectively (Figure 5A). Similarly, administering the vaccines via oral, IN, and IM routes resulted in a 1.6-fold, 2.2-fold, and 1.2-fold increase in MLN CD8^+^ T cell proliferation compared to control, each, respectively (Figure 5B). In both lymphoid organs, IN immunization elicited the strongest CD8^+^ T cell proliferation. Similar phenomenon was detected for GC B cell responses. Noticeable depreciation in GC B cells occurred in unimmunized mice upon *T. gondii* infection. This, however, did not occur in immunized mice (Figure 5C,D). In comparison to the Naïve+Cha, splenic GC B cell populations were enhanced by 1.6-fold, 2.3-fold, and 2-fold for the oral, IN, and IM routes, each respectively. Similarly, oral and IN immunization elicited an approximately 3.8-fold increase in GC B cells, whereas IM immunization resulted in a 3.2-fold increase. Overall, GC B cell induction was comparable across the three immunization groups. Scatter plots representing CD8^+^ T cell and GC B cell populations in MLN for all groups were provided (Figure 5E,F).

### 3.6. Pro-Inflammatory Cytokine Response in the Brain

**Figure 6:**
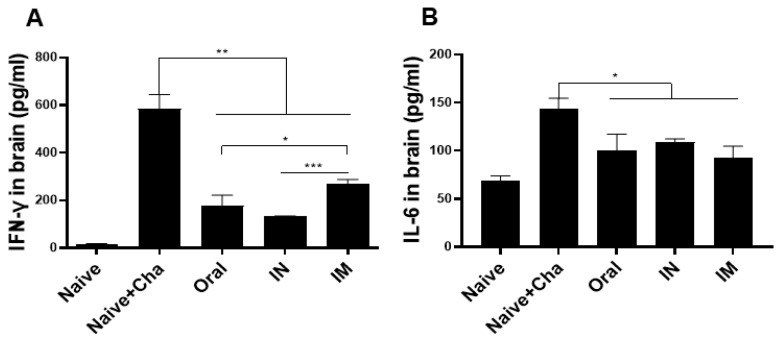
Brain tissues were collected from mice at 35 dpi with *T. gondii* ME49. The pro-inflammatory cytokines IFN-γ (**A**) and IL-6 (**B**) were measured from brain supernatants. Data are presented as mean ± SD and asterisks indicate statistical differences between groups. (* *p* < 0.1, ** *p* < 0.01, *** *p* < 0.001). All samples were acquired on an individual basis and a total of three independent experiments were performed in triplicates.

To evaluate the *T. gondii*-induced inflammatory response in the brain, mice were sacrificed at 35 dpi, and inflammatory cytokine production in the brain tissues was assessed. Compared to the naïve group, drastically high levels of IFN-γ were produced in Naïve+Cha group. Immunizing mice with the ROP18-expressing vaccines resulted in a nearly 3-fold reduction in IFN-γ production in the brain (Figure 6A). Reductions in IL-6 production, though significant, were not as dramatic as in the case of IFN-γ. Immunization, irrespective of the administration route, ensured that IL-6 production was retained near basal levels (Figure 6B).

### 3.7. Protective Efficacy of the Heterologous Immunization Strategy

**Figure 7:**
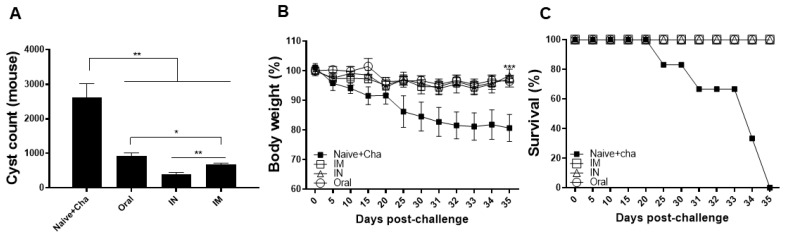
Cysts were isolated from the brain tissues of mice and counted under the microscope (**A**). Changes in body weight (**B**) and survival (**C**) were recorded daily until 35 dpi. Data are presented as mean ± SD and asterisks indicate statistical differences between groups. (* *p* < 0.05, ** *p* < 0.01, *** *p* < 0.001).

To confirm the protective efficacy of the vaccine against *T. gondii* ME49 infection, cysts in the brain homogenates were counted under the microscope. An average of 2500 cysts were detected from the Naïve+Cha group (Figure 7A). Substantially diminished cyst burden was observed in the brains of immunized mice. Compared to the Naïve+Cha, cyst reductions were 2.8-fold, 6.8-fold, and 3.9-fold for oral, IN, and IM immunizations, each, respectively. Consistent with this data, the body weights of mice were retained at normal levels (Figure 7B). Body weight reductions were negligible for the immunized mice, with none of the mice experiencing weight loss exceeding 5% of its initial value. In agreement with this data, 100% survival was observed until 35 dpi in the immunized mice. On the contrary, unimmunized mice gradually perished over time and none of the mice survived by 35 dpi (Figure 7C). These results suggest that heterologous immunization using rBVs-ROP18 and VLP-ROP18 vaccines is highly effective.

## 4. Discussion

Mounting of a strong mucosal immune response is paramount to limiting parasitic infestation. Here, we employed a heterologous immunization using rBVs and VLPs to evaluate the vaccination route-dependent protection in mice against *T. gondii*. Our results demonstrated that protection elicited by oral immunization against type II *T. gondii* strain is comparable to those induced by IM and IN immunization routes.

Heterologous immunization studies involving *T. gondii*, albeit limited, reported that this strategy was more effective than homologous immunization with various vaccine platforms. GRA7-expressing DNA-protein prime-boost regimen substantially enhanced protection in mice, compared to mice receiving either vaccine platform alone [19]. AMA1-expressing DNA-recombinant adenovirus prime-boost regimen also yielded similar results against *T. gondii* infection in mice [20]. Consistent findings were reported from other groups evaluating the vaccine efficacy of the DNA-virus vector prime-boost strategy in mice [21,22]. In line with this notion, there was a significant improvement in protection in this study, at least when compared to our previous study. *T. gondii* infection in ROP4 rBV-immunized mice resulted in drastic body weight fluctuation [11]. Yet, such findings were not detected in the present study and none of the immunized mice experienced body weight loss exceeding 5% upon challenge infection.

To date, only a handful of studies investigated the protection against *T. gondii* induced by ROP18 vaccines. However, much of our findings are in agreement with previous findings. The presence of Peyer’s patches and nasopharynx-associated lymphoid tissues for the two immunization routes are thought to have contributed to inducing mucosal immunity [23]. One study reported significant cyst burden reduction and intestinal IgA antibody induction by ROP18 DNA vaccine immunization [24]. As expected, and also in line with these reports, mucosal IgA response was detected from both oral and IN immunization groups in the present study. CD8^+^ T cell response is a crucial component of the host’s immune response that contributes to regulating both acute and chronic phases of the *T. gondii* infection [25]. Immunizing mice with the ROP18 DNA vaccine enhanced the activity of both CD4^+^ and CD8^+^ T cells, while also prolonging their survival following *T. gondii* RH infection [26]. Consistent with this report, heterologous immunization with ROP18-expressing rBVs and VLPs ensured that CD8^+^ T cells were maintained at sufficiently high levels. Interestingly, CD8^+^ T cell induction using the heterologous immunization strategy far exceeded those elicited upon homologous VLP immunization in mice. Multi-antigenic VLPs expressing ROP18 as one of the antigens merely restored the CD8^+^ T cells to basal levels or marginally increased it [16,17], but their proliferation was substantially enhanced by ROP18 rBV-VLP prime-boost strategy irrespective of administration route. Earlier studies have delineated the importance of *T. gondii*-specific IgA antibodies. Particularly, *T. gondii*-specific secretory IgA antibodies were reported to be crucial for inhibiting parasitic entry [27,28]. Given the profoundly high levels of parasite-specific mucosal IgA produced in the intestinal tract, it is reasonable to expect a certain degree of protection was mounted by oral immunization. Pro-inflammatory cytokine production within the central nervous system is a prominent feature observed during chronic *T. gondii* infection, and persistent neuroinflammation can lead to neuronal and psychiatric disorders [29]. Pro-inflammatory cytokine production was reduced following heterologous ROP18 vaccination, thus limiting the potential development of neuropathological symptoms.

Recently, one study revealed that parasitic dissemination into the brain can occur even at a low infection dose of 30 *T. gondii* ME49 cysts in C57BL/6 mice [30]. While the dose used in the aforementioned study better reflects natural *T. gondii* infection in animals, several factors must be taken into consideration. First, the genotype background of the mouse plays a strong role in the outcome of infection. The Th1-biased C57BL/6 are more susceptible to *T. gondii* infection than the Th2-biased BALB/c mice, which tend to be more resilient to *T. gondii*-induced encephalitis [31]. Furthermore, using a low infection dose is unsuitable in vaccine-related studies. Infecting mice with a low dose of moderately virulent ME49 strain is not likely to incur death in infected mice. Even if some of the mice perished, one cannot rule out the possibility that this was due to random chance and not the pathogen itself. To rule out this possibility, a lethal dose of 450 cysts was used for infection. Immunizing mice with the ROP18-expressing vaccines ensured 100% survival against this dosage, thus implying that our vaccines were effective. 

As anticipated, cyst reduction occurred to the greatest extent in the IN immunization group. To our surprise, despite failing to induce robust mucosal immunity, IM injection of ROP18 vaccines elicited greater cyst burden reduction than the oral immunization route. While it may appear that IM immunization induces greater protection in mice, findings here must be interpreted with caution. The high cyst count observed in the oral immunization group can be attributed to the severe antigen degradation occurring in the gastrointestinal tracts of mice, which is a major challenge for oral vaccines. Evidently, despite multiple immunizations, oral immunization elicited low levels of serum antibody response. On the contrary, IN and IM induced strong *T. gondii*-specific antibody responses. It is important to note that, even when affected by antigen degradation and diminished immunogenicity, cyst counts and overall protection were comparable between oral and IM groups. Furthermore, as the ultimate goal is to develop an effective *T. gondii* vaccine for human use, our findings must also consider how IM immunization affects humans, both physiologically and psychologically. In humans, the muscle mass at the site of injection and the needle length are some of the factors that affect the efficacy of needle-based IM vaccines [32]. For example, hepatitis B virus vaccine efficacy was reported to be substantially higher in human subjects immunized with longer needles [33,34]. IM injection with longer needles also incurred lesser side effects in children than with shorter needles [35,36]. Using long needles to maximize vaccine efficacy comes at the cost of patient discomfort, which can lead to trypanophobia and vaccine hesitancy. Ingestible oral vaccines are unlikely to cause discomfort or inflict pain, thereby promoting patient compliance with vaccination.

## 5. Conclusions

In summary, we evaluated the protective efficacy elicited upon heterologous immunization with the ROP18-expressing vaccine and attempted to determine the optimum immunization route. We found that the overall protection was comparable across all of the immunization routes used in the study, as they evoked complete protection against *T. gondii* ME49 strain in mice. Since oral vaccines are safe and easy to administer and convenient for all age groups, sequentially administering rBV and VLP vaccines by oral route demonstrated herein may be suitable for further development.

## Data Availability

The original contributions presented in this study are included in the article. Further inquiries can be directed to the corresponding author.
